# A Pilot Study on the Safety of a Novel Antioxidant Nanoparticle Delivery System and Its Indirect Effects on Cytokine Levels in Four Dogs

**DOI:** 10.3389/fvets.2020.00447

**Published:** 2020-07-30

**Authors:** Kelsey Robinson, Simon Platt, Katherine Bibi, Frane Banovic, Renee Barber, Elizabeth W. Howerth, Gary Madsen

**Affiliations:** ^1^Department of Small Animal Medicine and Surgery, College of Veterinary Medicine, University of Georgia, Athens, GA, United States; ^2^Department of Pathology, College of Veterinary Medicine, University of Georgia, Athens, GA, United States; ^3^ProTransit Nanotherapy, LLC, Omaha, NE, United States

**Keywords:** antioxidant, nanoparticle, spinal cord injury, catalase, superoxide dismutase, canine

## Abstract

Acute spinal cord injury consists of a primary, traumatic event followed by a cascade of secondary events resulting in ongoing cell damage and death. There is great interest in prevention of these secondary effects to reduce permanent long-term neurologic deficits. One such target includes reactive oxygen species released following injury, which can be enzymatically converted into less harmful molecules by superoxide dismutase and catalase. Canine intervertebral disc herniation has been suggested as a naturally occurring model for acute spinal cord injury and its secondary effects in people. The aims of this study were to test the safety of a novel antioxidant delivery system in four healthy dogs and to indirectly test effect of delivery via cytokine measurement. All dogs experienced adverse events to some degree, with two experiencing adverse events considered to be severe. The clinical signs, including combinations of bradycardia, hypotension, hypersalivation, pale gums, and involuntary urination, were consistent with complement activation-related pseudoallergy (CARPA). CARPA is a well-known phenomenon that has been reported to occur with nanoparticle-based drug delivery, among other documented causes. Two dogs also had mild to moderate changes in their blood cell count and chemistry, including elevated alanine transferase, and thrombocytopenia, which both returned to normal by day 7 post-administration. Cytokine levels trended downwards over the first 3 days, but many were elevated at measurement on day 7. Intradermal testing suggested catalase as a potential cause for reactions. No long-term clinical signs were observed, and necropsy results revealed no concerning pathology. Additional evaluation of this product, including further characterization of reactions to catalase containing components, dose-escalation, and desensitization should be performed before evaluation in clinically affected dogs.

## Introduction

Spinal cord injury (SCI) occurs with an estimated annual incidence of 15–54 cases per million people in developed countries (1–4). SCI most commonly affects young adult males, but there is an additional increase in occurrence in patients 65 years of age and older (1, 5). Despite progressive understanding and early recognition of disease, it remains devastating to those affected and has a large economic impact (2, 6).

Damage from SCI consists of a primary injury such as contusion, compression, or kinking of the cord that is commonly followed by a cascade of complex cellular and molecular events that leads to ischemia, inflammation, and potentially glial cell and neuronal death (2, 7, 8). The resulting cell damage following a primary mechanical injury is referred to as secondary SCI. One such mechanism of secondary SCI is activation of phagocytic inflammatory cells, primarily resident microglia, which leads to further inflammation and apoptosis, resulting in release of reactive oxygen species (ROS) such as superoxide (${\text{O}}_{2}^{-}$), hydroxyl radicals (OH–), and hydrogen peroxide (H_2_O_2_) (7, 9). These molecules contribute further to inflammation, cell damage, and apoptosis, leading to spread of injury (6, 7).

Though inflammatory cells are a large component of secondary injury, they are not the sole contributor to ROS presence following SCI, thus targeting reduction of ROS overall rather than a specific component of the cascade is of interest (9). Superoxide dismutase (SOD), an enzyme that catalyzes ${\text{O}}_{2}^{-}$ into O_2_ and H_2_O_2_, and catalase, an enzyme that degrades H_2_O_2_, are ideal therapeutics to reduce ROS. Unfortunately, due to a short half-life and poor uptake into neurons, exogenous delivery can be a challenge. Instead, one solution to delivery is via polylactic-co-glycolic acid (PLGA) nanoparticles (9–12).

Clinical SCI in dogs has been studied as a large animal model for human SCI, with similarities in mechanisms of injury, pathology, and outcome (13–16). An *ex vivo* evaluation of microglial cells in canine SCI found increased production of ROS from activated microglia in dogs affected by SCI (17), further supporting their utilization as an intermediate translational model for human secondary SCI.

The aim of our study was to evaluate the safety of a novel PLGA nanoparticle delivery system of SOD and catalase in healthy beagles. Our primary goal was to document physiologic effects throughout administration to determine whether the product was safe for clinical use. A secondary goal was indirect measurement of the efficiency of delivery via measurement of cytokines before and after infusion at 1, 3, and 7 days post-administration.

## Materials and Methods

### Nanoparticles

Pro-NP™ is a proprietary composition of NPs made up of the antioxidant enzymes SOD and catalase encapsulated in PLGA, a biodegradable polymer. The original formulation was developed at the University of Nebraska Medical Center (Omaha, NE), then two patents (US Patents 7,332,159 and 8,182,807) were licensed by UneMed Corp., the Center's technology transfer office, to ProTransit Nanotherapy, LLC (Omaha, NE). Average hydrodynamic diameter of Pro-NP™ in water measured using dynamic light scattering method was 152.4 nm with polydispersity index of 0.131 ± 0.005 and zeta potential of −1.41 mV. Size and zeta potential of Pro-NP™ were determined using a Zetasizer, Nano ZS, Malvern Panalytical.

### Study Design

Experimental design and study protocols were performed with the approval of the Institutional Animal Care and Use Committee (IACUC approval A2018 03-030-Y3-A6) at the University of Georgia. Four purpose-bred beagles were obtained and found to be clinically normal via physical examination, rectal temperature, heart rate, and respiratory rate. Neurologic examination was normal in all dogs. Dogs were premedicated with 2.2 mg/kg body weight of carprofen by mouth 12 h prior and 2 mg/kg body weight of diphenhydramine intramuscularly 15 min prior to the start of infusion. A 22 g intravenous catheter was placed in a cephalic vein following hair clipping and sterile preparation of the insertion site was performed with alcohol and chlorhexidine. The catheter was flushed with sterile heparinized saline prior to infusion. Dogs 1 and 2 received 6 mg/kg body weight of nanoparticles over a time span of 30 min. Dogs 3 and 4 received 6 mg/kg body weight over 60 min. Clinical evaluation of heart rate, respiratory rate, capillary refill time (CRT), and systolic blood pressure via Doppler evaluation of each dog took place every 5 min throughout the infusion time. Upon completion, a rectal temperature, heart rate, and respiratory rate were obtained every 30 min for the first 4 h, and was repeated at 8, 24 h, 3 and 7 days after the injection.

Blood was obtained via a sterile 22 g butterfly needle and a 5 ml syringe with sterile preparation of the draw site at baseline, 24 h post-, 3, and 7 days post-infusion. A complete blood count, serum chemistry, and bile acids were performed. Additionally, serum from these timepoints was evaluated on the Luminex Milliplex Canine panel of 13 cytokines. Cytokines evaluated included granulocyte macrophage colony stimulating factor (GM-CSF), interferon gamma (IFN-γ), keratinocyte chemotactic (KC)-like, IFN-γ induced protein (IP)-10, interleukin (IL)-2, IL-6, IL-7, IL-8, IL-10, IL-15, IL-18, monocyte chemotactic protein (MCP)-1, and tumor necrosis factor alpha (TNF-α). An important note is that IP-10 is also known as C-X-C motif chemokine ligand 10 (CXCL10), and MCP-1 is also known as C-C motif chemokine ligand 2 (CCL2).

### Intradermal Testing

Intradermal testing (IDT) for each individual component of the NP infusion was performed ~6 months post-nanoparticle infusion. Testing was performed in 4 dogs total, of which 3 received the original infusion, and a fourth that was naïve to the product and unlikely to be primed for an IgE mediated immune response. A large (~15 × 10 cm) area for injection of the test substances was clipped on the right and left lateral thoracic region 24 h prior to the testing. This 24-h delay prevented a skin reaction or irritation from clipping to interfere with the test. Dogs were sedated with 5 μg/kg dexmedetomidine (Domitor, Pfizer, Exton, PA) intravenously; it has been established that medetomidine does not interfere with intradermal skin testing in dogs (18, 19). A volume of 0.05 ml per testing solution was administered intradermally via a 1 ml allergy syringe with a 27-gauge needle. Solutions tested included dimethyl tartrate (DMT) 0.05 and 0.1 mg/mL solution, SOD 0.1 and 0.2 mg/mL solution, catalase 0.1 and 0.2 mg/mL solution, Pro-NP Blank 0.5 and 1 mg/mL solution, Pro-NP SOD 0.5 and 1 mg/mL solution, Pro-NP Catalase 0.5 and 1 mg/mL solution, human serum albumin (HSA) 0.05 and 0.1 mg/mL solution, blank (PBS; negative control), and a 0.1 mg/mL histamine phosphate control (positive control). The concentrations of the test substances were determined by maximum total concentration in the final product as recommended by the developer.

The order of injections was randomized using statistical computer software (Prism 8.0) by assigning numbers to each component. Injections were prepared by one investigator (KR) and administered by a separate investigator (FB), who was blinded to the contents of each syringe and gave injections based on numerical label only. Thirty minutes after each injection, the extent and severity of the wheals were assessed by the same investigator (FB) who performed injections.

Global wheal score (GWS) was calculated from all measurements as previously reported: GWS = D × E × F (the average diameter (D) in orthogonal directions was measured in millimeters; erythema (E) and firmness (F) (20–22).

### Histopathology

Approximately 6 months post-infusion, all dogs that underwent intradermal testing were euthanized via an overdose (200 mg/kg body weight) of pentobarbital and submitted for necropsy. Histopathology was performed for all dogs, with the IDT naïve dog serving as a control comparison for dogs that had received the NP infusion. Pieces of lung, liver, spleen, and kidney were removed and immersed in 10% buffered formalin, processed routinely for embedding in paraffin, and 4 μ sections were stained with hematoxylin and eosin.

### Statistical Analysis

Due to small sample size, no statistical analyses were performed.

## Results

### Animals

Four purpose-bred beagles were obtained. There were two males and two females, all unaltered with an age range from 2 to 7 years and weights between 12 and 16 kgs.

### Adverse Events

Adverse events of varying severity were present in all dogs and are described below:

Dog 1 experienced pale pink mucous membranes 10 min into the infusion. It was quickly followed by onset of bradycardia (50 bpm) identified by palpation of pulses and confirmed by cardiac auscultation. Heart rate returned to a normal range (90–100 bpm) over 5 min with no intervention. She remained alert, interactive, and standing with no hypersalivation, urination, or hypotension.

Dog 2 vomited bile 8 min into the infusion. At 10 min, he developed pale to white mucous membranes, began hypersalivating, and had difficulty standing, which progressed to lateral recumbency and involuntary urination. Doppler evaluation found severe systolic hypotension of 40 mmHg and the infusion was paused for a 1/3 shock bolus of crystalloids. The heart rate remained at 150 bpm and respiration was normal at 28 breaths per minute throughout. Ten minutes after the start of the bolus, blood pressure normalized to 110 mmHg, mucous membranes returned to normal pink color, and patient was able to easily stand. The infusion was resumed and completed with no further events.

Dog 3 developed weakness, hypersalivation, and pale mucous membranes 10 min into the infusion. He was hypotensive on Doppler at 50 mmHg and progressed to laterally recumbent followed by involuntary urination. Heart and respiratory rate remained stable at 120 bpm and 32 breaths per minute, respectively. The infusion was paused but upon starting a crystalloid bolus, the catheter became non-patent. During the time of placement of a new catheter, blood pressure improved to 80 mmHg and strength and mucous membrane color returned to normal. Repeat blood pressure was 100 mmHg. The infusion was resumed with no additional concerns.

Dog 4 experienced pale pink mucous membranes 9 min into the infusion. She developed lip-licking with no overt hypersalivation. There was mild expiratory effort with a normal rate (32 breaths per minute) and normal auscultation. Heart rate remained at 120 bpm and all signs resolved within 5 min of onset. No weakness, urination, or hypotension occurred.

### Clinical Pathology

All abnormal values are listed in Table 1. All values for all time points are available in the Supplemental Materials.

**Table 1:** Abnormal blood values for all dogs at all time points with reference ranges. Any values within range are marked with “–.”

	**Value**	**Normal range**	**Baseline**	**24-h**	**3 day**	**7 day**
Dog 1	Red blood cells	5.90–8.66 × 10^∧^6/ul	4.57 (L)	4.41 (L)	4.51 (L)	4.70 (L)
	Hemoglobin	13.7–20.7 g/d	10.6 (L)	10.2 (L)	10.2 (L)	10.8 (L)
	Hematocrit	l42.2–59.8%	30.3 (L)	29.5 (L)	30.1 (L)	31.0 (L)
	RBC distribution width	11.2–13.2%	14.2 (H)	14.3 (H)	14.4 (H)	14.5 (H)
	Platelets	226–424 × 10^∧^3/ul	438 (H)	–	–	430 (H)
	Plateletcrit	0.15–0.45%	0.47 (H)	–	0.47 (H)	0.53 (H)
	Chloride	106–114 mmol/L	104 (L)	–	–	104 (L)
	Bicarbonate	16–25 mmol/L	26 (H)	–	–	–
	Urea nitrogen	9–28 mg/dl	7 (L)	7 (L)	–	8 (L)
	Creatinine	0.6–1.3 mg/dl	0.5 (L)	0.5 (L)	0.5 (L)	0.4 (L)
	Cholesterol	109–345 mg/dl	512 (H)	441 (H)	402 (H)	393 (H)
Dog 2	RBC distribution width	11.2–13.2%	14.8 (H)	14.6 (H)	14.8 (H)	15.0 (H)
	Platelets	226–424 × 10^∧^3/ul	–	167 (L)	222 (L)	–
	Bicarbonate	16–25 mmol/L	26 (H)	–	–	–
	Alanine transferase	12–106 U/L	–	–	109 (H)	–
	Albumin	3.3–4.2 g/d	–	–	–	4.3 (H)
Dog 3	Red blood cells	5.90–8.66 × 10^∧^6/ul	–	–	5.86 (L)	–
	Hemoglobin	13.7–20.7 g/d	–	–	12.9 (L)	–
	Hematocrit	l42.2–59.8%	–	41.2 (L)	38.1 (L)	40.3 (L)
	RBC distribution width	11.2–13.2%	14.9 (H)	14.8 (H)	15.3 (H)	15.4 (H)
	Platelets	226–424 × 10^∧^3/ul	–	–	189 (L)	–
	PDW	39.9–67.2%	39.3 (L)	–	–	–
	Total Protein	5.4–7.1 g/dl	7.2 (H)	–	–	–
	Globulins	2.7 g/dl 1.8–3.0	3.5 (H)	3.1 (H)	–	–
	Albumin/globulin ratio	1.2–2.0	1.1 (L)	1.1 (L)	–	–
	Alanine transferase	12–106 U/L	–	192 (H)	133 (H)	170 (H)
	Calcium	9.5–11.2 mg/dl	–	9.3 (L)	–	–
	Magnesium	1.8–2.4 mg/dl	–	–	–	1.7 (L)
Dog4	Mean corpuscular HGB concentration	33.0–35.0 g/dl	–	–	–	35.4 (H)
	Red blood cells	5.90–8.66 × 10^∧^6/μl	–	5.48 (L)	5.46 (L)	–
	Hemoglobin	13.7–20.7 g/d	–	13.2 (L)	13.4 (L)	–
	Hematocrit	l42.2–59.8%	–	38.1 (L)	38.5 (L)	–
	RBC distribution width	11.2–13.2%	14.3 (H)	14.4 (H)	14.4 (H)	15.1 (H)
	Plateletcrit	0.15–0.45%	–	–	–	0.55 (H)
	MPC	17.2–23.6 g/dl	–	–	–	16.9 (L)
	Segmented neutrophils	2.700–8.500 × 10^∧^3/μl	–	–	–	8.9 (H)
	Urea nitrogen	9–28 mg/dl	8 (L)	–	8 (L)	8 (L)
	Bile acids	0.0–5.0 μmol/L	–	–	5.4 (H)	–

#### Complete Blood Count

Dog 1 had a persistent mild anemia and a mildly elevated RBC distribution width (RDW) throughout the study. These changes were present from baseline and remained largely unchanged throughout the study. A thrombocytosis and elevated plateletcrit (PCT) was present at baseline and day 7. Dog 2 had a persistently mildly elevated RDW and a mild thrombocytopenia at 24 h and on day 3. Dog 3 had a persistently mildly elevated RDW, and a low platelet distribution width (PDW) on day 1. A mild anemia was present at 24, day 3, and day 7. A thrombocytopenia was present at day 3. Dog 4 had a persistently mildly elevated RDW throughout the study. There was a mild anemia at 24 h and day 3. At day 7, there was a mild increase in mean corpuscular hemoglobin concentration (MCHC) and PCT, and a decrease in mean platelet component (MPC). Segmented neutrophils were also mildly increased.

Though only two subjects developed thrombocytopenia, the mean reduction in platelets among the group was 82.25 × 10^3^/ul from baseline to 24 h and 52 × 10^3^/ul from baseline to day 3 post-administration. All dogs recovered to within normal range by day 7, and no clinical signs associated with thrombocytopenia were noted at any time point.

#### Blood Chemistry

Dog 1 had persistent mild to moderate hypercholesterolemia from baseline throughout the study, as well as decreased renal values including creatinine (creat) and blood urea nitrogen (BUN). There was mild hypochloremia at baseline and day 7. Bicarbonate was elevated at baseline, but values were within normal range at the rest of the sampling times. Dog 2 had an elevated bicarbonate at baseline that was not present at other times. Alanine transferase (ALT) was slightly elevated at day 3 and albumin (ALB) was slightly elevated at day 7. Dog 3 had mild hyperproteinemia with a hyperglobulinemia and low albumin/globulin (A/G) ratio at baseline. The hyperproteinemia resolved, but mild hyperglobulinemia was present at 24 h. There was a mild total hypocalcemia at 24 h and no other time point. There was mildly to moderately elevated ALT at 24 h post-, 3, and 7 days post-administration. Mild hypomagnesemia was present at day 7. Dog 4 had a mildly decreased BUN at baseline, 3, and day 7. Bile acids were slightly high on day 7.

#### Cytokine Analysis

**Figure 1:**
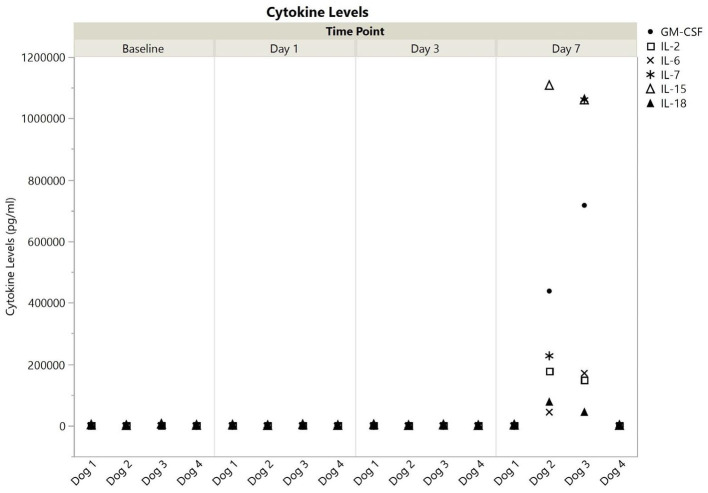
Cytokine levels (pg/ml) with at least one measurement at >100,000 for dogs 1–4 at baseline, 1, 3, and 7 days post-nanoparticle administration. GM-CSF, granulocyte-monocyte colony stimulation factor; IL, interleukin.

**Figure 2:**
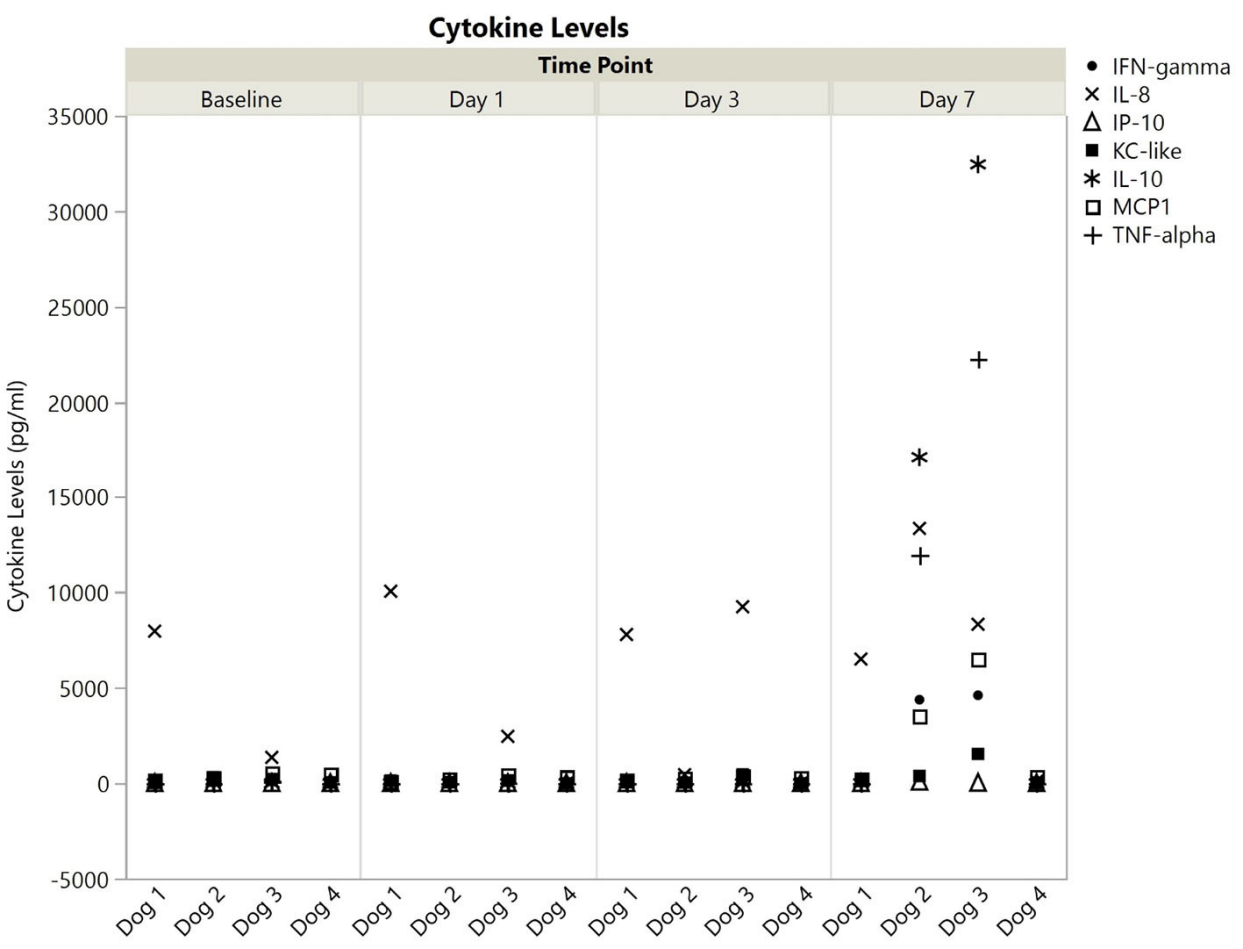
Cytokine levels (pg/ml) with all values <100,000 for dogs 1–4 at baseline, 1, 3, and 7 days post-nanoparticle administration. IFN, interferon; IL, interleukin; IP, IFN-γ induced protein; KC, keratinocyte chemotactic; MCP, monocyte chemotactic protein; TNF, tumor necrosis factor.

All cytokines were detected in at least one dog at one timepoint, and all values are available in the Supplementary Material. Cytokine levels, broken down into those with or without a single measurement above 100,000 pg/ml are shown for each dog at each time point in Figures 1, 2. Published ranges for healthy controls were used for consideration of normal vs. out of range, though there are no strict agreements on normal levels in dogs (23, 24). There was no detectable IP-10, IL-10, or TNF-α at any timepoint for dogs 1 and 4. Dog 1 had no detectable GM-CSF, IL-2, IL-6, or IL-18 at any time point, and all other cytokine measurements were within reported ranges for healthy dogs. Additionally, dog 4 did not have any measurements outside of reported ranges. Interestingly, in all detectable cytokines except IFN-γ as compared to values as baseline, levels at 1 and day 3 initially trended downwards until day 7.

In contrast to dogs 1 and 4, dogs 2, and 3 had more profound cytokine changes noted especially at day 7. There were multiple cytokines which required further dilution (1:10) as they were beyond readable limits at initial dilution (1:3). These included GM-CSF and IL-15 for both dogs, and IL-2, IL-7, and IL-18 for dog 3. Even following a 1:10 dilution, an IL-15 level could not be obtained for dog 3, so the highest registered value was used as the final point.

#### Intradermal Testing

Intradermal testing scores are available in Table 2. Intradermal injection of histamine resulted in a positive wheal and erythema reactions in all dogs. There were no wheal and flare reactions observed after the intradermal injections of PBS (negative control).

**Table 2:** Global wheal scores for all solutions with positive reactions to intradermal injections.

	**Positive Control (histamine−0.1 mg/ml)**	**Negative Control (PBS)**	**Catalase 0.1 mg/ml**	**Catalase 0.2 mg/ml**	**Pro-NP Catalase 0.5 mg/ml**	**Pro-NP Catalase 1 mg/ml**
Dog 1	103.5	7.0	7.0	7.0	7.0	7.5.0
Dog 2	99.0	7.5	23.0	50.0	7.0	22.0
Dog 3	130.5	7.0	40.0	40.0	99.0	99.0
Dog 4^*^	126.0	8.0	7.0	22.0	8.0	8.0

* *dog 4 in this study is different than original recipient of NP infusion. This dog served as a naïve subject compared to pre-exposed dogs*.

Dog 1 did not react to any of the test substances. Dog 2 developed erythematous wheals after intradermal injection of catalase 0.1 mg/mL and 0.2 mg/mL solution and the Pro-NP catalase 1 mg/mL solution. Dog 3 showed erythematous wheals to the catalase 0.1 mg/mL and 0.2 mg/mL solutions and to the Pro-NP catalase at 0.5 mg/mL and 1 mg/mL. Dog 4 showed only erythematous wheals to the catalase 0.2 mg/mL solution.

#### Histopathology

Histopathology did not reveal any changes suggestive of toxicity in the parenchymal organs. Within the liver there was mild hepatocellular lipofuscinosis in 4/4 dogs, scattered hemosiderin laden macrophages in 2/4, and rare canalicular plugs in 1/4 dogs. There was mild extramedullary hematopoiesis and scattered hemosiderin laden macrophages in the spleen of 4/4 dogs. The kidneys had mild multifocal medullary mineralization in 4/4 dogs and there was mild osseous metaplasia in the lungs of 1/4 dogs. Findings in the liver, spleen, and lungs are common age-related changes and were considered incidental. Medullary mineralization (4/4) was also considered an incidental change which was likely diet related. There was no difference in the dogs which received NPs and the dog that did not.

## Discussion

One of several key players in secondary SCI is oxidative stress due to the presence of highly reactive free radicals, which are largely comprised of ROS such as superoxide ($O_{2}^{-}$) and hydroxyl radicals (OH^−^) (25–27). Under typical conditions, superoxide is converted to hydrogen peroxide (H_2_O_2_) by SOD, which is then converted to O_2_ and H_2_O via catalase (2, 8).

$$\begin{array}{l} \cdot O_{2\;}^{-}\;\overset{superoxide\;dismutase}{\to }\;H_{2}O_{2\;}\overset{catalase}{\to }O_{2}+H_{2}O \end{array}$$

When there is free iron present in addition to H_2_O_2_, hydroxyl radicals can be formed by Fenton's reaction (25, 26, 28).

The primary sources of ROS following SCI are activated microglia and leukocytes (macrophages and neutrophils), which typically gain entry to the affected site via vascular disruptions from primary SCI (9, 27, 29). Almost immediately following primary injury, endogenous antioxidants are depleted, shifting to a pro-oxidant and pro-inflammatory environment (27, 28, 30). ROS react with polyunsaturated fats [such as arachidonic acid, linoleic acid, eicosapentaenoic (EPA) acid, and docosahexaenoic acid (DHA)] via lipid peroxidation, leading to disruption of phospholipid-dependent enzymes, disruption of ionic gradients, and possible membrane lysis when severe enough. The inflicted cell damage leads to further release of free radicals and thus cell damage spreads from the epicenter of the primary injury in a feedback loop (8, 27, 28).

Therapies aimed at reduction of secondary injury are of particular interest as a way to prevent the cascade of events leading to cell death. Administration of exogenous antioxidant enzymes has historically been difficult due to their short half-life and the fact that because of their inherent negative charge at physiologic pH, they do not readily cross cell membranes. Even in injuries leading to transient disruption of the blood brain barrier which allows these enzymes to cross, they do not seem to be taken up by astrocytes or neurons (9, 11, 31). In an effort to combat these challenges, our group has developed a PLGA construct which encapsulates the antioxidant enzymes SOD and catalase for delivery following traumatic spinal cord injury. PLGA is an FDA approved polymer which is biocompatible and biodegradable (32). Additionally, it can allow for sustained release of contents, which may be beneficial for ongoing inflammatory processes in secondary SCI. In an *in vitro* evaluation of human neurons, the catalase-loaded PLGA NPs were found to provide neuroprotection from H_2_O_2_ induced cytotoxicity (12). In a rat reperfusion injury model, PLGA NPs loaded with both catalase and SOD mitigated the inflammatory response, prevented apoptosis of neuronal cells, and protected the blood brain barrier from ROS-mediated reperfusion injury as compared to blank NPs (10). Intravenous administration to rat and pig models of SCI found a dose-dependent localization of NPs to the site of injury, and in rats, the NPs could be detected at the site of epicenter even at 4 weeks post-injury (33). The dose for our study (6 mg/kg) was determined based on these previous publications.

When given to this group of dogs, all exhibited adverse effects to varying degrees and for brief periods during nanoparticle infusion. Two dogs displayed only a mild cardiovascular change of bradycardia that resolved within 5 min of onset with no intervention. The remaining two dogs experienced more severe cardiovascular and neurologic changes, including weakness, hypersalivation, involuntary urination, and severe hypotension. Both recovered within 15 min of onset with one recovering following a crystalloid bolus and the other without any intervention. The first severe reaction occurred in a dog receiving the infusion over 30 min, and the second was in a dog receiving it over 60 min. The acute reactions observed were most consistent with the reported syndrome of complement activation-related pseudoallergy (CARPA), which can occur with administration of intravenous nanoparticle enhanced drugs, protein-based drugs, or radiocontrast agents (34–36). CARPA is a well-documented occurrence in medicine, with clinical signs similar to those of an allergic hypersensitivity reaction; however, in contrast to classic hypersensitivity reactions, CARPA reactions occur upon initial exposure, decrease in severity with repeated exposure, and can resolve spontaneously (35, 37, 38).

Dogs with suspected CARPA reactions exhibit systemic hyper- or hypotension, profound but reversible blood cell changes including leukopenia with subsequent leukocytosis and thrombocytopenia, autonomic neurologic changes (hypersalivation, urination, defecation), and, rarely, cutaneous flushing (39). These clinical signs are the result of a cascade of events: complement activation leads to release of anaphylatoxins (primarily C3a and C5a) which activate blood cells and other allergy-mediator secretory cells to release vasoactive inflammatory mediators which then cause autonomic responses such as capillary leakage, bronchoconstriction, vasoconstriction, and vasodilation. The mediators released from these cells can then go on to recruit and further activate additional cells, leading to a cycle of physiologic abnormalities (35, 40). The degree to which a patient is affected can vary from mild to severe, with fatality reported in some cases. Regarding PLGA specifically, an evaluation of PLGA NP interaction with blood components, including complement, found that PLGA NPs on their own were very weak activators of the complement protein C3 (5%), but activation increased with changes in encapsulation products (20–25%) and more so with surface functionalization (40–60%) (41). Given an amplification signal in the complement cascade, one theory is that small changes to initial activation could lead to more prominent clinical signs similar to those that were demonstrated in our dogs (42). Additionally, several studies have found that CARPA appears to be dose-dependent in nature, so potential dose reduction may reduce the reactions we observed (43, 44).

A major challenge in CARPA is the fact that there is no reliable way to predict reactions prior to administration (45). While there are assays for complement products and cytokines for humans, the presence of a positive reaction with a nanomedicine can only identify risk of reaction without accurate prediction of severity or incidence (46). For drugs with a known reaction, patients are commonly premedicated with corticosteroids and antihistamines to prevent potential adverse events (34, 35, 47). These drugs may also be started at a low infusion rate and only titrated upwards if patients are not exhibiting any symptoms of a reaction. Alternatively, in the case of severe reactions, treatment may be stopped and then attempted again at a lower dose after receiving additional corticosteroids (48).

In previous evaluations of this specific nanoparticle infusion in rats, there were no reported episodes of suspected CARPA; however, rats have been found to be two to three orders of magnitude less sensitive to these types of reactions when compared with pig, which are a common CARPA model. Other evaluations have found that pigs, dogs, and humans appear to have approximately similar sensitivity, so reactions within our patients may be more representative of possible risk in human subjects (39, 49, 50). However, it is important to consider that the suspected primary initiator of these reactions in pigs is the pulmonary intravascular macrophage (PIM)—a cell which is unlikely to play a role in dogs, rats, or healthy humans (46, 51–53). Instead, the cell suspected to play the largest role in species other than pigs are resident macrophages in the liver and spleen, though this concept remains unvalidated (46, 49).

In our case, we did not obtain blood until 24 h following nanoparticle administration; thus, we did not see any changes to white blood cell counts as have been reported immediately following administration (39). We did, however, see other bloodwork changes in the two dogs that reacted the most severely. Dog 2 developed a mild ALT elevation on day 3 and had a mild thrombocytopenia at 24 h and 3 days, both of which resolved by day 7. Dog 3 developed a persistent mild ALT elevation by 24 h and a transient thrombocytopenia by day 3. Activation of Kupffer cells via complement and resulting inflammatory mediators may explain elevations in ALT due to hepatocyte damage, and thrombocytopenia is suspected to be due to consumption during the inflammatory response. No dog exhibited any clinical signs beyond those during administration and necropsy results 6 months post-administration did not reveal any permanent pathology to liver, lungs, kidneys, or spleen in any dog receiving the infusion.

To attempt to elucidate the cause of reactions observed in our dogs, intradermal skin testing was performed for each of the individual components of the final product administered, including two concentrations of each DMT, SOD, catalase, blank Pro-NPs, SOD loaded Pro-NPs, catalase loaded Pro-NPs, HSA, PBS (negative control), and histamine phosphate (positive control). Interestingly, the only positive reactions noted were for catalase alone or catalase containing nanoparticles. It is important to recognize the limitation in this evaluation given the different mechanisms underlying an immunoglobulin (Ig) E mediated hypersensitivity vs. non-IgE mediated pseudoallergy, and that intradermal testing may not be reliable in detection or prediction of CARPA (35, 40). However, this method has been explored in human medicine, particularly in relation to radiocontrast agents either following a known reaction or as a screening tool. As a testing method in known reactors, it was found to be 96% specific (54). Unfortunately, there are mixed results on its use as a screening tool, with one study finding a 57.1% sensitivity for severe reactors (55), and another finding a 0% sensitivity and positive predictive value (56). Regardless, additional testing such as administration of the infusion without the catalase component or a dose escalation test would be needed to further investigate these suspicions prior to administration in a clinical setting.

Evaluation with a Luminex-based multiplex panel revealed significant elevations in almost all cytokines tested in dogs 2 and 3, and less overt changes in dogs 1 and 4. However, for detectable cytokines in dog 4, there was a decrease in levels at days 1 and 3 before a rise in values again at day 7. In dogs 2 and 3, there was also a trend downwards in most cytokines prior to the vast increase at day 7. This could potentially represent an initial benefit of the antioxidant enzymes on baseline cytokine levels. Unfortunately, values beyond 7 days were not obtained for evaluation of continued changes. The lack of cytokine elevation initially supports our suspicion of a complement-mediated rather than an IgE-based reaction upon administration of the nanoparticles, as one would expect higher cytokine levels immediately if it had been allergy mediated (57). It is unclear why there are only elevations at day 7, but a possible explanation could be that following infusion, the nanoparticles were processed by antigen presenting cells resulting in release of inflammatory mediators as would occur in a typical infection. Given the lack of clinical signs and poor characterization of normal cytokine levels, it is difficult to determine the relevance of these findings without further investigation.

One concern in utilization of enzymes to reduce ROS damage is the injury timeline. Secondary SCI begins almost immediately following a primary insult, so a rapid response is necessary to induce neuroprotection. However, the cutoff for time to administration is poorly defined. One study in rats with experimentally induced SCI found ROS levels approximately twice the basal level upon immediate sampling, a sustained elevation for over 10 h post-injury, and then a decline back to basal level by 12 h post-injury (25). Results of the second National Spinal Cord Injury Study (NASCIS-II), found that long term motor function recovery following traumatic SCI was improved among patients receiving methylprednisolone (30 mg/mg bolus followed by 5.4 mg/kg infusion for 23 h) as compared to naloxone or placebo if given within 8 h of injury, with prevention of lipid peroxidation as one of several proposed mechanisms (58, 59). It is important to note that the benefits of this treatment regimen have been a topic of debate within the medical community, with some repeat evaluations finding no benefit and others finding weak evidence for its use through systematic review (60–62). Similar studies in dogs have been performed, with comparable variation in results. In an experimentally induced SCI model, dogs receiving a combination of electroacupuncture and corticosteroids recovered faster than those receiving either treatment alone, which all recovered faster than the control dogs (63). A more recent prospective, randomized, blinded trial comparing paraplegic dogs with absent nociception (*n* = 58) receiving methylprednisolone sodium succinate (MPSS), polyethylene glycol (PEG), or placebo found no improved recovery in any group when administered within 24 h of onset of paralysis (64). This study further highlights the challenges of rapid interference, as only 21 dogs presented within 6 h of onset over a 5 year recruitment period. In a systematic review on recovery of non-ambulatory dogs, the time to presentation ranged from 0 to 305 days across several studies, with a total mean of 7.9 days for reported values (range 0.75–19 d) (65). Considering the common delay of presentation of affected dogs, the time to administration following injury is a limitation to this therapy. Regardless, there is a clear role for early oxidative stress in inducing cell damage and, thus, the sooner therapy can be initiated, the better (27).

## Conclusion

There is a vital role for prevention of ROS production following an acute SCI. PLGA nanoparticle delivery of SOD and catalase could provide sustained release of antioxidants following an acute SCI, mitigating the cascade of events following primary injury. Given the reactions that occurred during infusion in our dogs, additional testing including a dose escalation test, elimination from or reformulation of catalase in the product, complement protein measurement, alternative premedication strategies, and desensitization prior to full dose could all be explored to better classify the areas which need addressed prior to moving forward.

## Data Availability Statement

All datasets presented in this study are included in the article/Supplementary Material.

## Ethics Statement

The animal study was reviewed and approved by the University of Georgia Institutional Animal Care and Use Committee.

## Author Contributions

SP and GM contributed conception and design of the study. KR, SP, and FB carried out experimental procedures. KR organized the data and wrote the first draft of the manuscript. FB wrote the intradermal testing methods of the manuscript. EH performed all pathological evaluation. All authors contributed to manuscript revision, read, and approved the submitted version.

## Conflict of Interest

At the time of publication GM was the president and CEO of ProTransit Nanotherapy. Nanoparticles and individual components were provided by the company. The remaining authors declare that the research was conducted in the absence of any commercial or financial relationships that could be construed as a potential conflict of interest.
